# Results based on 124 cases of breast cancer and 97 controls from Taiwan suggest that the single nucleotide polymorphism (SNP309) in the MDM2 gene promoter is associated with earlier onset and increased risk of breast cancer

**DOI:** 10.1186/1471-2407-9-13

**Published:** 2009-01-13

**Authors:** Ying-Fang Sun, Jyh-Der Leu, Su-Mei Chen, I-Feng Lin, Yi-Jang Lee

**Affiliations:** 1 Department of Biomedical Imaging and Radiological Sciences, National Yang-Ming University, Taipei, Taiwan; 2 Division of Radiation Oncology, Taipei City Hospital Ren Ai Branch, Taipei, Taiwan; 3 Division of Nuclear Medicine, Taipei City Hospital Ren Ai Branch, Taipei, Taiwan; 4 Department of Public Health, National Yang-Ming University, Taipei, Taiwan

## Abstract

**Background:**

It has been suggested that the single nucleotide polymorphism 309 (SNP309, T -> G) in the promoter region of the MDM2 gene is important for tumor development; however, with regards to breast cancer, inconsistent associations have been reported worldwide. It is speculated that these conflicting results may have arisen due to different patient subgroups and ethnicities studied. For the first time, this study explores the effect of the MDM2 SNP309 genotype on Taiwanese breast cancer patients.

**Methods:**

Genomic DNA was obtained from the whole blood of 124 breast cancer patients and 97 cancer-free healthy women living in Taiwan. MDM2 SNP309 genotyping was carried out by restriction fragment length polymorphism (RFLP) assay. The multivariate logistic regression and the Kaplan-Meier method were used for analyzing the risk association and significance of age at diagnosis among different MDM2 SNP309 genotypes, respectively.

**Results:**

Compared to the TT genotype, an increased risk association with breast cancer was apparent for the GG genotype (OR = 3.05, 95% CI = 1.04 to 8.95), and for the TG genotype (OR = 2.12, 95% CI = 0.90 to 5.00) after adjusting for age, cardiovascular disease/diabetes, oral contraceptive usage, and body mass index, which exhibits significant difference between cases and controls. Furthermore, the average ages at diagnosis for breast cancer patients were 53.6, 52 and 47 years for those harboring TT, TG and GG genotypes, respectively. A significant difference in median age of onset for breast cancer between GG and TT+TG genotypes was obtained by the log-rank test (p = 0.0067).

**Conclusion:**

Findings based on the current sample size suggest that the MDM2 SNP309 GG genotype may be associated with both the risk of breast cancer and an earlier age of onset in Taiwanese women.

## Background

A functional single nucleotide polymorphism has been identified at position 309 within the first intron of the promoter region of the human *MDM2 *gene and hence designated SNP 309 [1]. Conversion of the T allele to the G allele in the region causes a higher affinity for the Sp1 transcription activator and subsequently enhances the transcription of MDM2 gene. Over-expression of MDM2 oncoprotein may result in a higher risk of carcinogenesis and accelerated tumorigenesis by negatively regulating p53 tumor suppressor protein [2].

Supporting evidence for the hypothesis that MDM2 SNP309 influences tumor formation is derived from the clinical outcomes obtained from different research groups. The median age of cancer diagnosis was found to be nine years younger in SNP309 carriers compared with non-carriers in Li-Fraumeni syndrome patients, including those with breast cancer (29 yrs v.s. 39 yrs, p = 0.01) [1]. MDM2 SN309 was further reported to accelerate sporadic breast cancer formation in Caucasians women in a gender-specific and hormone-dependent manner [3]. A similar observation was reported by analyzing a large cohort of familial breast cancer cases in South-West Netherlands, although the estrogen signaling and mutant BRCA1/2 or CHECK 1100delC was not required [4]. On the other hand, MDM2-SNP309 was reported to be associated with early onset of breast and ovarian cancer among BRCA1/2 mutant carrier of Jewish-Ashkenazi descent [5]. Interestingly, a recent report showed that in the Chinese population, MDM2 SNP309 G allele increased the risk of sporadic breast cancer, but the T allele was associated with earlier onset [6]. On the contrary, a combined study of 11 related breast cancer reports worldwide showed no effect of MDM2 SNP309 on the risk of breast cancer [7]. Moreover, a large pooled series of more than 5,000 cases from five European studies within the Breast Cancer

Association Consortium (BCAC) also concluded that no association could be found between MDM2 SNP309 and breast cancer [8]. However, both large cohort-based studies were unable to exclude MDM2 SNP309 involvement in breast cancer time of onset. Two very recent studies separately focusing on Indian women and Scottish Caucasian women also suggested no detectable association between MDM2 SNP309 and breast cancer, although this genetic polymorphism was associated with high grade nodal positive breast cancer in the latter population [9,10]. Due to conflicting findings, further elucidation of the role of MDM2 SNP309 in the risk of breast cancer and its time of onset as well as possible association with racial/ethnic subgroups is necessary.

In this study, we adopted a hospital-based case-control study to investigate the association between MDM2 SNP309 and onset of breast cancer in the female Taiwanese population. The first reason for this study was that relevant information on the effect of the MDM2 SNP309 on the risk of breast cancer remains sparse for Asian populations, including Taiwanese. Secondly, given that MDM2 SNP309 may influence tumor development in the selected patient subgroup, the regional epidemiological study was highly relevant and should provide important information from which to better understand the role of MDM2 SNP309 on breast cancer formation.

## Methods

### Research subjects

The participants were domestic Taiwanese women from Taipei City Hospital Ren Ai branch in Taipei, Taiwan. One hundred and twenty-four patients with sporadic breast cancers diagnosed by cancer specialists and 97 cancer-free healthy adult volunteers signed the consent form and filled out the structured questionnaire to participate this study. Other clinically defined metabolic symptoms such as cardiovascular diseases or diabetes were also inquired from the study subjects since it has been reported that these factors may be associated with breast cancer, including in the Taiwanese population [11-13]. Institutional review board (IRB) approval from National Yang-Ming University (IRB number: 970048, see additional file 1) was obtained for this study. The age range was 21 to 76 years (median 54 years) for breast cancer patients and 18 to 77 years (median 38 years) for healthy controls, respectively. Both of them were randomly and consecutively enrolled. Except MDM2 SNP309 genotyping, no other genetic background related to cancer development was obtained from all participants.

### MDM2 SNP309 genotyping

The genomic DNA preparation was performed on collected blood samples and the genotyping procedure was carried following a previous report with only minor modifications [14]. In brief, the genomic DNA was extracted from 200 μl of blood sample donated by the participants using the Qiagen mini blood DNA extraction kit (Qiagen Inc., Valencia, CA). The chromosome region containing the *MDM2 *SNP 309 was amplified by polymerase chain reaction using a pair of primers, namely F (5'-CGGGAGTTCAGGGTAAAGGT-3') and R (5'-AGCAAGTCGGTGCTTACCTG-3'). The PCR reaction consists of 100 ng of genomic DNA, 0.2 μM primer, 200 μM dNTP, 1.5 mM MgCl_2_, 20 mM Tris-HCl (_P_H 8.4), 50 mM KCl and one unit of Platinum Taq DNA polymerase (Invitrogen, Carlsbad, CA). The thermal cycler conditions were 94°C for one minute; 40 cycles with denaturing at 94°C, annealing at 58°C, and elongation at 72°C for 30 seconds each; one cycle at 72°C for 10 min. For restriction fragment length polymorphism (RFLP) analysis, 10–20 μl of the amplified 352 bp fragment was digested with one unit MSPA1I restriction enzyme (New England Biolabs, Ipswich, MA) at 37°C in a water bath for 30–60 minutes. The T/T, T/G and G/G genotypes were distinguished by observing the presence of fragments with lengths of 233 bp/88 bp, 233 bp/187 bp/88 bp, and 187 bp/88 bp after electrophoresis on a 3% ethidium bromide stained NuSieve agarose gel. The genotypes were further confirmed by direct sequencing of the PCR products by the Sequencing Core Facility of National Yang-Ming University Genomic Research Center (YMGC).

### Statistical analysis

The genotype and allele frequency of MDM2 SNP309 was tested for Hardy-Weinberg equilibrium using an on-line public statistical tool http://www.genes.org.uk/software/hardy-weinberg.shtml, and the statistical significance was determined by χ^2 ^test. Two-sample t tests and Chi-square tests were used to explore the bivariate association between the status of breast cancer and other covariates for continuous and categorical variables, respectively. Risk association between the genotypes and breast cancer development was estimated by odds ratio (OR) and 95% confidence intervals (CI) using multivariate logistic regression analysis. The variables shown to be statistically significant in the bivariate analysis were adjusted in the multivariate analysis. Among the breast cancer cases, Kaplan-Meier curves and the log-rank test were used to compare the median onset age between patients with GG and those with TG+TT genotypes in MDM2 SNP309. All statistical analyses were performed with Statistical Analysis System software (ver. 9.1; SAS Institute, Cary, NC).

## Results

The breast cancer patients (n = 124) and healthy controls (n = 97) were all native Taiwanese women. The characteristics of the healthy controls and breast cancer patients, including average ages, body mass index (BMI), breastfeeding experience, with or without high calorie intake, oral contraceptives usage, and history of cardiovascular disease/diabetes were summarized in Table 1. Among these characteristics, age, BMI, patients with cardiovascular diseases and diabetes, and oral contraceptives usage were significantly different between cases and controls after the statistical testing (see materials and methods). These confounding factors were adjusted in multivariate logistic regression analysis.

**Table 1:** The general characteristics of breast cancer patients and healthy controls from Taiwanese women blood donors

Characteristics, *n *(%)^*a*^	Cases	Control	*p*-value^*b*^
**Age (yrs)**			
Mean	53.3	38.7	< 0.0001
S.D.	9.62	15.2	
**Body mass index (BMI, kg/m^2^)**	24.09 ± 4.2	21.66 ± 3.13	< 0.0001
			
**Breastfeeding**			
Yes	53 (42.7)	33 (34)	0.2794
No	71 (57.3)	64 (66)	
**High calorie intake**			
Yes	34 (27.4)	31 (32)	0.2786
No	90 (72.6)	66 (68)	
**Cardiovascular disease/diabetes**			
Yes	42 (33.9)	15 (15.5)	0.0019
No	82 (66.1)	82 (84.5)	
**Oral contraceptive pills usage**			
Yes	31 (25)	11 (11.3)	0.0202
No	93 (75)	86 (88.7)	

*a*. Cases: n = 124; controls: n = 97. The percentages were shown in the parentheses

*b*. Age and BMI were determined by two-sample *t *tests; breastfeeding, high calorie intake, cardiovascular disease/diabetes, and oral contraceptive drugs usage were determined by chi-square tests.

The genotype and allele frequencies of MDM2 SNP309 calculated from 124 breast cancer cases and 97 controls of Taiwanese women were summarized in Table 2. Although the genotype frequency of the controls conformed to the Hardy-Weinberg equilibrium (χ^2 ^= 2.63), the breast cancer cases and the combined cohort showed a statistically significant difference between the observed and expected frequencies of the MDM2 SNP309 genotypes (χ^2 ^= 10.84 and 12.18, respectively). The frequencies of both heterozygous and homozygous genotypes were higher for breast cancer cases than for healthy controls (64.5% v.s. 58% for TG, and 21% v.s. 16% for GG). This observation suggests that the G allele in MDM2 SNP309 is associated with the risk of breast cancer in Taiwanese women. Furthermore, using SNP309TT as a reference, the age-adjusted odds ratios (ORs) for the TG and GG genotypes were 2.24 (95% CI, 0.97 to 5.19) and 3.40 (95% CI, 1.21 to 9.57), respectively (Table 3). When the ORs were further adjusted for age, BMI, cardiovascular diseases/diabetes, and oral contraceptives usage, they still showed a risk association of GG (OR = 3.05, 95% CI = 1.04 to 8.95) and TG (OR = 2.12, 95% CI = 0.90 to 5.00) genotypes in breast cancer cases (Table 3). Together, these data indicate that the homozygous MDM2 SNP309 GG genotype may increase the risk of breast cancer in Taiwanese women.

**Table 2:** MDM2 SNP309 genotype and allele frequencies of Taiwanese breast cancer cases and healthy controls

	MDM2 SNP309^*a*^		
	
Frequency	Cases, *n *(%)	Controls, *n *(%)	Entire cohort *n *(%)^*b*^
**Genotype**			
TT	18 (14.5)	25 (25.8)	43 (19.4)
TG	80 (64.5)	56 (57.7)	136 (61.7)
GG	26 (21)	16 (16.5)	42 (18.9)
TG+GG	106 (85.5)	72 (74.2)	179 (80.6)
			
**Allele**			
T	0.468	0.541	0.498
G	0.532	0.459	0.502

^a^The χ^2 ^of cases, controls and entire cohort are 10.84, 2.63, and 12.18, respectively. Degree of freedom (*d.f*.) = 2

^b ^Entire cohort is the sum of each genotype in breast cancer cases and healthy controls.

**Table 3:** The risk evaluation of MDM2 SNP309 genotypes on the development of breast cancer in Taiwanese women

SNP309^*a*^	OR (95% CI)^*b*^	*p*-value	OR (95% CI)^*c*^	*p*-value	OR (95% CI)^*d*^	*p*-value
TT	1		1		1	
TG	1.98 (1.00 to 3.98)	0.05	2.24 (0.97 to 5.19)	0.06	2.12 (0.90 to 5.00)	0.08
GG	2.26 (0.95 to 5.38)	0.07	3.40 (1.21 to 9.57)	0.02	3.05 (1.04 to 8.95)	0.04
TG+GG	2.06 (1.05 to 4.06)	0.04	2.48 (1.10 to 5.60)	0.03	2.30 (1.00 to 5.30)	0.05

*a *The frequency of each genotype is referred to Table 2.

*b *The crude OR values.

*c *Age-adjusted OR values.

*d *These OR values are the results adjusted for age, BMI, cardiovascular disease/diabetes, and OCP (Oral Contraceptive Pill) usage

**Figure 1:**
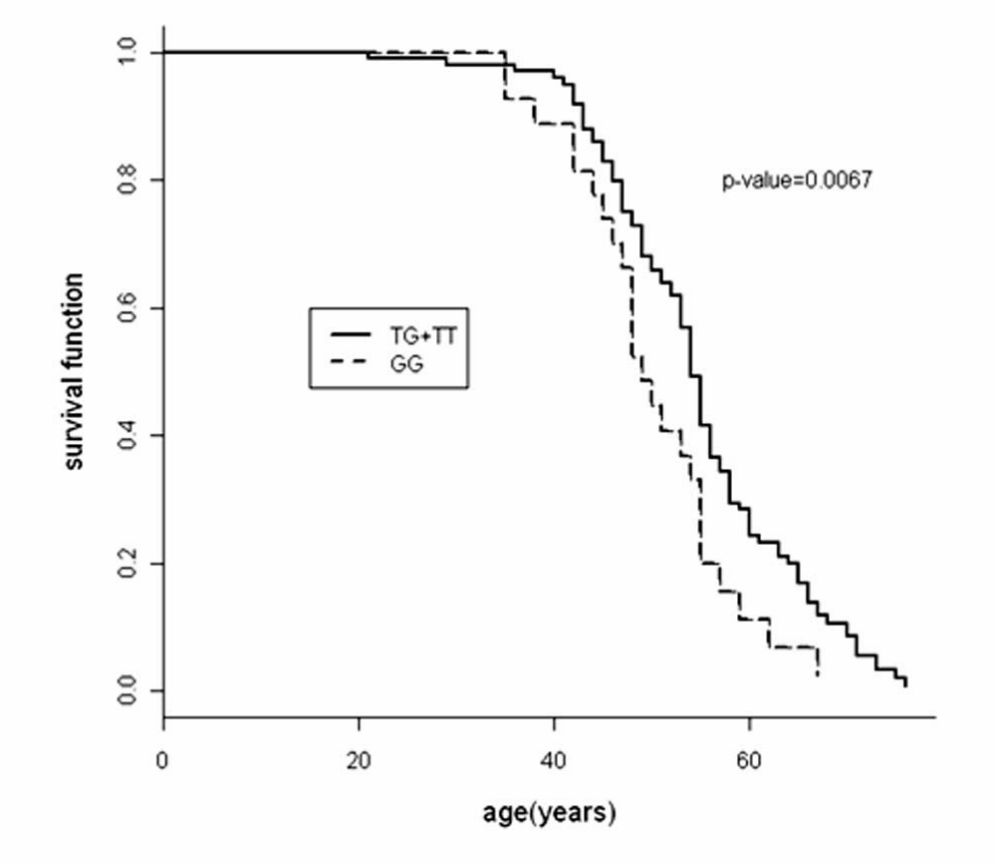
**Association between MDM2 SNP309 GG genotype with earlier age onset of breast cancer cases in Taiwanese women**. The survival curve of GG genotype was compared with that of TG+TT genotype. The Y axis represented the cumulative case-free survival rate against age at diagnosis. The statistical difference between two survival curves was evaluated by log-rank test.

Among the breast cancer patients, the mean ages at diagnosis for the TT, TG and GG genotypes of MDM2 SNP309 were 53 (range 42–66), 51.5 (range 21–72) and 46.9 (range 30–64) years old, respectively. The survival analysis using the Kaplan-Meier method showed that the onset age of breast cancer in patients with GG genotypes and TG+TT genotypes was significantly different (log-rank test, p = 0.007, Figure 1). Therefore, current data suggest that homozygous GG but not TG genotype of MDM2 SNP309 may also accelerate the incidence of breast cancer among the female Taiwanese population.

## Discussion

In this study, we found that MDM2 SNP309 was associated with the risk of breast cancer incidence as well as an earlier onset of this disease in female Taiwanese population (124 breast cancer cases). Compared to other reports, such as the large-cohort studies of BCAC in Europeans (5,191 cases) and in African-Americans and Caucasians in North Carolina (2,037 cases) [8,15], or studies with relatively smaller sample sizes in Japanese (557 cases), Chinese (366 cases), British (351 cases), Germany (549 cases), Americans in Baltimore (294 cases), Turkish (223 cases), Indian (104 cases), and Scottish (299 cases) [9,10,16-21], more cases from Taiwanese women are expected to be assessed in the future. Nevertheless, our result is consistent with the assumption that certain subgroups may be affected by this genetic background on tumor development [8,18,22].

In addition to small sample size, this hospital-based case-control study has several limitations. For instance, ages and BMI of breast cancer participants were significantly older and higher than that of the healthy controls in this study. However, all of the samples were consecutive participants who consented to be enrolled in this study, and it was not possible to predict the genotype of MDM2 SNP309 among them. In addition, we adjusted these confounding factors in subsequent statistical analyses to minimize the potential biasing effects. Another limitation is that other genetic factors, such as p53 or BRCA1/2 genotypes have not been assessed in our study. Therefore, it cannot be ruled out that MDM2 SNP309 may combine with these genetic factors to affect the development of breast cancer in Taiwanese women. Indeed, Huang et al. has found that an interaction between MDM2 SNP309 and mutant p53 is able to affect tumor behavior in sporadic oral squamous cell carcinomas in Taiwanese [23]. To the best of our knowledge, a similar interaction has not been studied in the cases of Taiwanese breast cancer yet. Taken together, increased sample size and investigation of other genetic factors will be important in better understanding the effects of MDM2 SNP309 on breast cancer development in the Taiwanese population.

The association of metabolic syndromes and lifestyle to breast cancer was also taken into account in our study. High calorie diet usually leads to obesity, hyperglycemia, hyperinsulinemia, diabetes mellitus and hypertension [13,24]. Increase incidence of breast cancer in the Asian population has in part paralleled the shift toward Western dietary practices that cause the development of obesity [25-27]. It has been reported that Taiwanese patients with diabetes have higher risk of breast cancer mortality at all ages [11]. Also, high dietary fat intake as well as usage of oral contraceptives significantly increase the risk of breast cancer in Taiwanese women [28,29]. On the contrary, breast feeding may exert a protective effect in the Taiwanese population [30]. In addition to the Taiwanese-based studies, these potential factors in the risk assessment of breast cancer have also been evaluated by other groups worldwide [12,13,31-38]. Accordingly, the patients with cardiovascular disease/diabetes and oral contraceptive usage were adjusted for multivariate logistic regression analysis since these factors exhibited significant differences among cases and controls by Chi-square tests. It will be of interest to further investigate whether it is possible for an interaction between MDM2 SNP309 and obesity-associated metabolic syndromes to affect breast cancer by assessing a larger Taiwanese population.

It is unknown whether the rate of the MDM2 SNP309 GG genotype (21%) among Taiwanese breast cancer patients is significantly different from other ethnic groups. When we compared this frequency to other ethnic groups with breast cancer, the results were as follows for the various studied populations: Anglo-Saxon (16.8%), Chinese (23.2%), Indian (30%), German (16%), Caucasian (18.4%), African-American (0.6%), Jewish-Ashkenazi (33%) and South-West Netherlands (14%) [4,9,15,17,18]. This showed that the Taiwanese and the Chinese patients exhibited most similar frequency of GG genotype compared to other ethnic groups. Ma et al., have reported that G allele of MDM2 SNP309 may be not involved in the risk of breast cancer formation from Jiangsu province in mainland China (OR = 0.92, 95% CI = 0.62 to1.37) [18]. However, Lum et al. demonstrated that the MDM2 SNP309 G allele increased risk in sporadic breast cancer in a Chinese population from Shanghi city [6]. Given that the main immigrants of Taiwan are from mainland China, this implies that the effects of MDM2 SNP309 on the risk of breast cancer may be associated with environmental discrepancy and confined to the selected subgroups even in similar ethnicities.

Similar to the risk estimation, whether MDM2 SNP309 is associated with an earlier age onset of breast cancer remains controversial [1,4,17,18,39]. Using Kaplan-Meier survival analysis, we found that breast cancer patients with GG genotype were 5 to 6 years earlier in age at diagnosis compared to TT+TG genotype in Taiwanese women. Such a result partially agrees with the report by Bond et al., which showed that GG women are associated with a 7-year average earlier age of onset of breast cancer compared with TT women [39]. However, whether such a relationship is associated with the expression level of the estrogen receptor is unknown from this study. On the other hand, this observation conflicts to the report claiming that T allele is essentially associated with earlier onset of breast cancer in the Chinese population [6]. It is speculated that MDM2 SNP309 may play a complex role in the time of onset for breast cancer among various subgroups.

Taken together, this hospital-based case-control study suggests that MDM2 SNP309 is associated with the risk of breast cancer in Taiwanese women. Furthermore, the GG genotype can accelerate the age at diagnosis compared to the TT+TG genotype. The small sample size of this study may limit the conclusion ascribed from our statistic assessment. Nevertheless, smaller sample size has also been reported in findings on the effects of MDM2 SNP309 on breast cancer and endometrial cancers [1,14]. A perspective study that is based on a larger sample size is necessary, and it will be important to evaluate the interaction between MDM2 SNP309 and other genetic backgrounds to identify the significance of MDM2 SNP309 on the risk and onset time of breast cancer in the Taiwanese population.

## Conclusion

Our data suggested that the homozygous MDM2 SNP309GG genotype simultaneously affected the risk and the onset age of breast cancer in the Taiwanese population. Given that several reports showed no association between MDM2 SNP309 and the risk of breast cancer, our findings suggest that such a polymorphism is possibly dependent on various subgroups. A larger sample size is expected to assess the association between MDM2 SNP309 and breast cancer incidence in Taiwanese women in the future.

## Competing interests

The authors declare that they have no competing interests.

## Authors' contributions

Y-FS was responsible for DNA extraction, MDM2 SNP309 genotyping and other administrative works. J-DL and S-MC were responsible for collection of blood samples, signed consent forms and filled questionnaires. I-FL performed the statistical analysis and provided interpretation of the results. Y-JL designed the study, carried out the genotyping, investigated the progression of this study and prepared the manuscript. This final manuscript has been read and approved by all co-authors.

## Pre-publication history

The pre-publication history for this paper can be accessed here:

http://www.biomedcentral.com/1471-2407/9/13/prepub

## Supplementary Material

Additional file 1 **IRB approval document.** An approval document of institutional review board. Click here for file
