# Supplementary figures and images for: Results based on 124 cases of breast cancer and 97 controls from Taiwan suggest that the single nucleotide polymorphism (SNP309) in the MDM2 gene promoter is associated with earlier onset and increased risk of breast cancer
Source: BMC Cancer. 2009 Jan 13;9:13. doi: 10.1186/1471-2407-9-13 (PMC2649157; doi:10.1186/1471-2407-9-13)

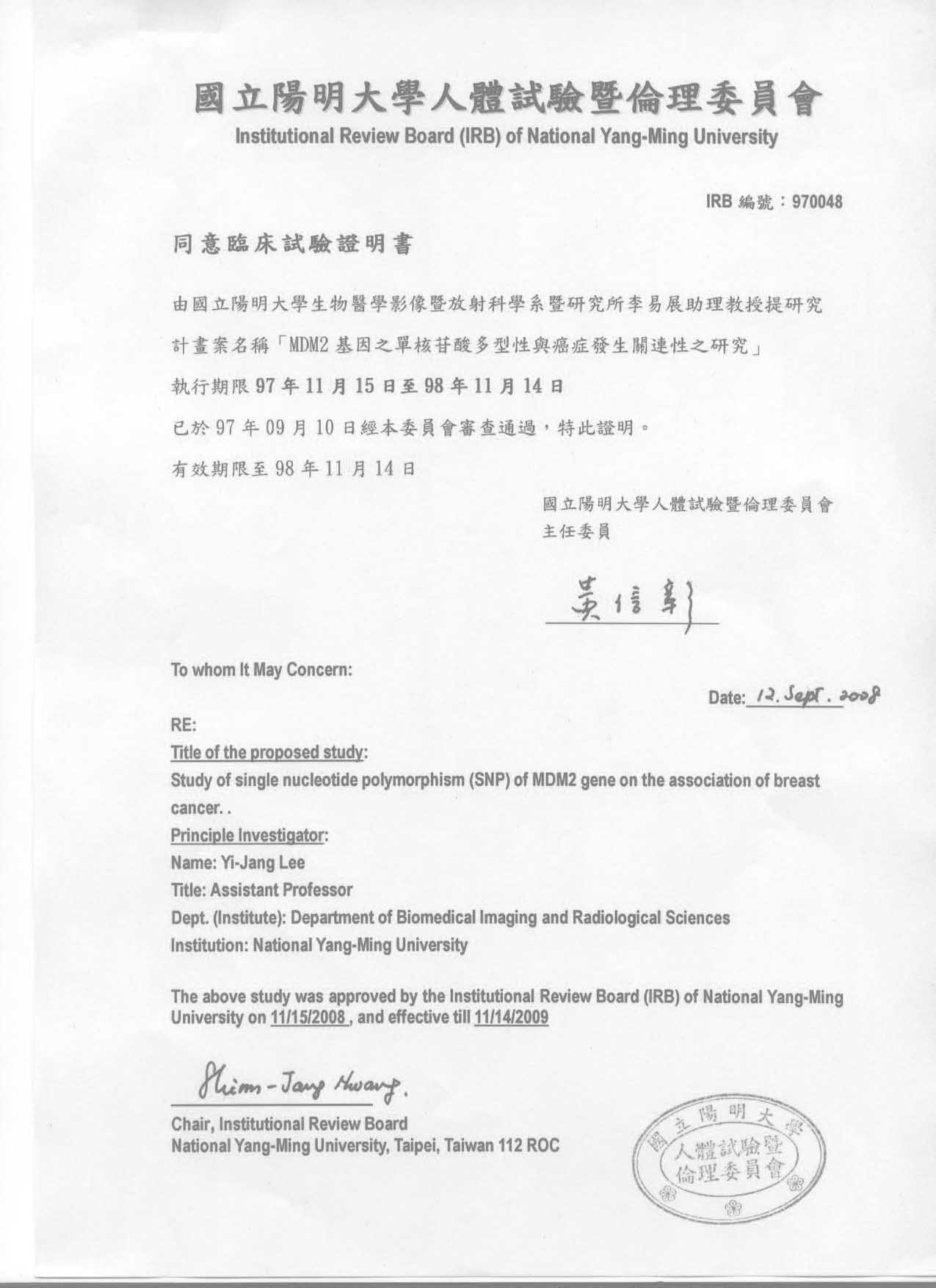

Supplement: Additional file 1 — IRB approval document. An approval document of institutional review board. [file 1471-2407-9-13-S1.jpeg]
